# Human NK cells adapt their immune response towards increasing multiplicities of infection of *Aspergillus fumigatus*

**DOI:** 10.1186/s12865-018-0276-6

**Published:** 2018-12-18

**Authors:** Lothar Marischen, Anne Englert, Anna-Lena Schmitt, Hermann Einsele, Juergen Loeffler

**Affiliations:** Department of Internal Medicine II, WÜ4i, University Hospital Wuerzburg, Wuerzburg, Germany

**Keywords:** *Aspergillus fumigatus*, Aspergillosis, NK cells, Chemokines, CCL4, MIP-1β, Multiplicity of infection

## Abstract

**Background:**

The saprophytic fungus *Aspergillus fumigatus* reproduces by generation of conidia, which are spread by airflow throughout nature. Since humans are inhaling certain amounts of spores every day, the (innate) immune system is constantly challenged. Even though macrophages and neutrophils carry the main burden, also NK cells are regarded to contribute to the antifungal immune response. While NK cells reveal a low frequency, expression and release of immunomodulatory molecules seem to be a natural way of their involvement.

**Results:**

In this study we show, that NK cells secrete chemokines such as CCL3/MIP-1α, CCL4/MIP-1β and CCL5/RANTES early on after stimulation with *Aspergillus fumigatus* and, in addition, adjust the concentration of chemokines released to the multiplicity of infection of *Aspergillus fumigatus*.

**Conclusions:**

These results further corroborate the relevance of NK cells within the antifungal immune response, which is regarded to be more and more important in the development and outcome of invasive aspergillosis in immunocompromised patients after hematopoietic stem cell transplantation. Additionally, the correlation between the multiplicity of infection and the expression and release of chemokines shown here may be useful in further studies for the quantification and/or surveillance of the NK cell involvement in antifungal immune responses.

**Electronic supplementary material:**

The online version of this article (10.1186/s12865-018-0276-6) contains supplementary material, which is available to authorized users.

## Background

As a mold fungus, *Aspergillus (A.) fumigatus* is part of our everyday environment. Naturally living in the soil, *A. fumigatus* is capable of colonizing dead plants, rotting wood, and also wet areas often frequented by humans, as for example cellars or swimming pools. Within its reproductive cycle, *A. fumigatus* generates spores (“conidia”), that are easily distributed by air flow [1]. Therewith, humans often inhale certain amounts of spores per day [2]. Fungal pathogens are recognized by the innate immune system via pattern recognition receptors such as Toll-like receptors (TLRs), c-type lectin receptors (CLRs), complement receptor 3 and galectin family proteins, and subsequently damaged by neutrophils and/or finally phagocytosed by alveolar macrophages. Since dendritic cells may get involved, different subgroups of T cells will eventually contribute to the immune response [3]. Nevertheless, *A. fumigatus* can bring on allergies like asthma or allergic bronchopulmonary aspergillosis (ABPA) [4]. Furthermore, immunocompromised patients in general, and – increasingly encountered in clinical practice – patients after hematopoietic stem cell transplantation (HSCT), are severely endangered to develop invasive aspergillosis (IA) after *A. fumigatus* infection [5]. The recovery of the immune system after HSCT starts with the appearance of innate immune cells such as granulocytes, monocytes and dendritic cells within the first weeks. Natural killer (NK) cells are the first lymphoid cells to show up in peripheral blood, and their numbers are reciprocally correlated with the severity of IA [6, 7].

NK cells are cluster of differentiation (CD)56^+^ CD3^−^ lymphocytes originally characterized by their ability to arrange apoptosis of virus-infected or neoplastic cells without a previous sensitization process. Up to now, NK cells or adequate subsets were found in several tissues of the human body such as lungs, liver, skin, intestine, uterus, bone marrow, spleen, lymph nodes [8], blood, decidua [9], or central nervous system [10]. In bronchoalveolar lavage fluid, macrophages account for more than 80% of total immune cells, while NK cells constitute just around 1% [11]. Even though this hardly suggests a major contribution of NK cells, several studies lay special emphasis on the fact that NK cells still account for 10% of the lymphocytes in the lung, while they additionally show a higher percentage of differentiated/matured cells than in other peripheral organs such as liver, skin and secondary lymphoid tissues. The fast recruitment of additional NK cells just hours (h) after the onset of inflammation may be supported by the regular dynamic movement of NK cells between blood and lung tissue, that leaves just a very small subpopulation of tissue-resident CD69^+^ NK cells as required for immune surveillance [8, 12, 13]. It is therefore tempting to speculate, that the low amount of NK cells initially present in the lung can increase very rapidly when needed, and subsequently contribute substantially to the immune response.

It is still under discussion, whether the contribution of NK cells to the immune response against pathogens is strongly dependent on accessory cells [14] or can be fully or in part explained by their expression of pattern recognition receptors like TLRs and nucleotide oligomerization domain (NOD)-like receptors [15]. In this context, Chalifour et al. showed a TLR-dependent release of α-defensin by highly purified NK cells [16]. Expression of other peptides with antimicrobial characteristics, for example X-C motif chemokine ligand 1 (XCL1)/lymphotactin, lysozyme, granulysin, α-defensin 6 [17], perforin [18] and cathelicidin/LL-37 [19], was reported.

Further studies have characterized the integration of NK cells within the cytokine network of the immune system. NK cell functions are affected by several interleukins (IL) as IL-1, IL-10, IL-12, IL-15 and IL-18 [20], and by chemokines such as CC chemokine ligand (CCL)2/monocyte chemoattractant protein (MCP)-1, CCL3/macrophage inflammatory protein (MIP)-1α, CCL4/MIP-1β, CCL5/regulated and normal T cell expressed and secreted (RANTES), CCL10/N-gamma-inducible protein-10 (IP-10), CCL19/MIP-3β, CCL21/ secondary lymphoid tissue chemokine (SLC) and chemokine (C–X3–C motif) ligand 1 (CX3CL1)/fractalkine [21]. Recruitment of NK cells is mediated by CCL3/MIP-1α, CCL4/MIP-1β, CCL5/RANTES, CCL19/MIP-3β, CCL21/SLC, CXCL8/IL-8, CXCL10/IP-10, CXCL11, CXCL12/stromal cell-derived factor 1 and CX3CL1/fractalkine [21, 22]. By themselves, NK cells can produce tumor necrosis factor-α (TNF-α) and interferon-γ (IFN-γ) most prominently, as well as IL-5, IL-10, IL-13, granulocyte-macrophage colony-stimulating factor (GM-CSF), CCL2/MCP-1, CXCL8/IL-8, CXCL10/IP-10, XCL1/lymphotactin, CCL1 and CCL22/ macrophage-derived chemokine [20, 21]. CCL3/MIP-1α, CCL4/MIP-1β and CCL5/RANTES were found early on in the supernatant of stimulated NK cells [20].

In conclusion, NK cells are present at the typical gateway for fungal infections, are expressing pattern recognition receptors and antimicrobial peptides, and are involved in the cytokine network of the immune system. Recent reviews from Schmidt et al. [23] and Ogbomo and Mody [24] summarize current knowledge about the interaction of NK cells with fungal pathogens in general. Concerning *A. fumigatus*, the essential role of NK cells is underscored by animal studies in neutropenic mice [25, 26] and in vitro experiments pointing to a critical role of IFN-γ and perforin [27, 28]. CD56 [29], NKp30 [30] and NKp46 [31], which are prototypic surface molecules for NK cells, are reported as binding receptors for fungal ligands. Interestingly, the morphotype of *A. fumigatus* is critical for the onset of an immune response by NK cells. As published by Schmidt et al. and our study group, respectively, germlings and hyphae induce fungicidal activity, while conidia seem to pass unnoticed [27, 28]. In this respect, Aimanianda et al. have shown the rodlet/hydrophobin layer on *A. fumigatus* conidia to prevent immune recognition [32]. It appears to be obvious, that this rodlet/hydrophobin layer becomes fragile during the germination and growing processes of the fungus, and therefore exposes immunogenic structures below. On the other hand, *A. fumigatus* actively interact with lung epithelial cells, macrophages, neutrophils and components of the complement system [33]. As well, *A. fumigatus* impairs NK cell-derived immune functions by downregulating the levels of GM-CSF and IFN-γ in the supernatant of pre-stimulated NK cells [28, 34].

Here, we aimed to characterize the expression and release of immunomodulatory molecules by NK cells early on after stimulation with *A. fumigatus*, and accessed the effects of a varying multiplicity of infection (MOI). Thereby, this study contributes to the understanding of the interaction between NK cells and *A. fumigatus*, which is regarded to be more and more important in the progression of fungal infections in immunocompromised patients recovering from HSCT.

## Results

### Cytokine expression levels in NK cells after *A. fumigatus* stimulation

**Fig. 1:**
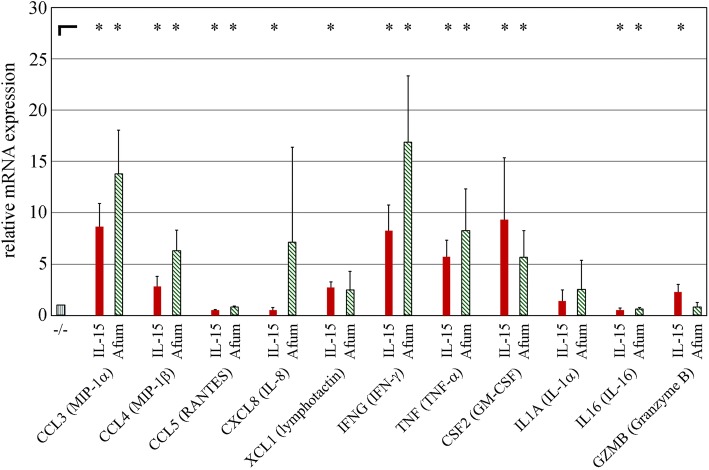
Screening for cytokine mRNA expression by quantitative PCR. Freshly isolated NK cells were pre-incubated overnight with pro-leukin and stimulated with IL-2 /IL-15 (IL-15) or *A. fumigatus* (Afum) germ tubes for 6 h. RNA was isolated and analyzed for the relative expression of the indicated genes by qPCR. Results were normalized for the value of unstimulated NK cells (−/−) – which was specified as “1”. *n* = 5, means and standard deviations are shown. *p < 0.05. Please note CCL5/RANTES, which was significantly downregulated in a very small range

In a first approach, expression of selected cytokines was screened by quantitative polymerase chain reaction (qPCR) analysis of NK cells after stimulation with *A. fumigatus*. Therefore, freshly isolated NK cells were pre-incubated overnight with IL-2 and subsequently stimulated with IL-2/IL-15 or *A. fumigatus* germ tubes for 6 h. Unstimulated controls were included. Ribonucleic acid (RNA) was isolated and used for qPCR screening analysis (Fig. 1). As shown, the expressions of the chemokines CCL3/MIP-1α, CCL4/MIP-1β, CXCL8/IL-8, XCL1/lymphotactin and the cytokines IFN-γ, TNF-α, GM-CSF and IL-1α were increased due to stimulation by *A. fumigatus* or – as for granzyme B – just by IL-2/IL-15. CCL5/RANTES and IL-16 expressions, on the other hand, were significantly downregulated.

**Fig. 2:**
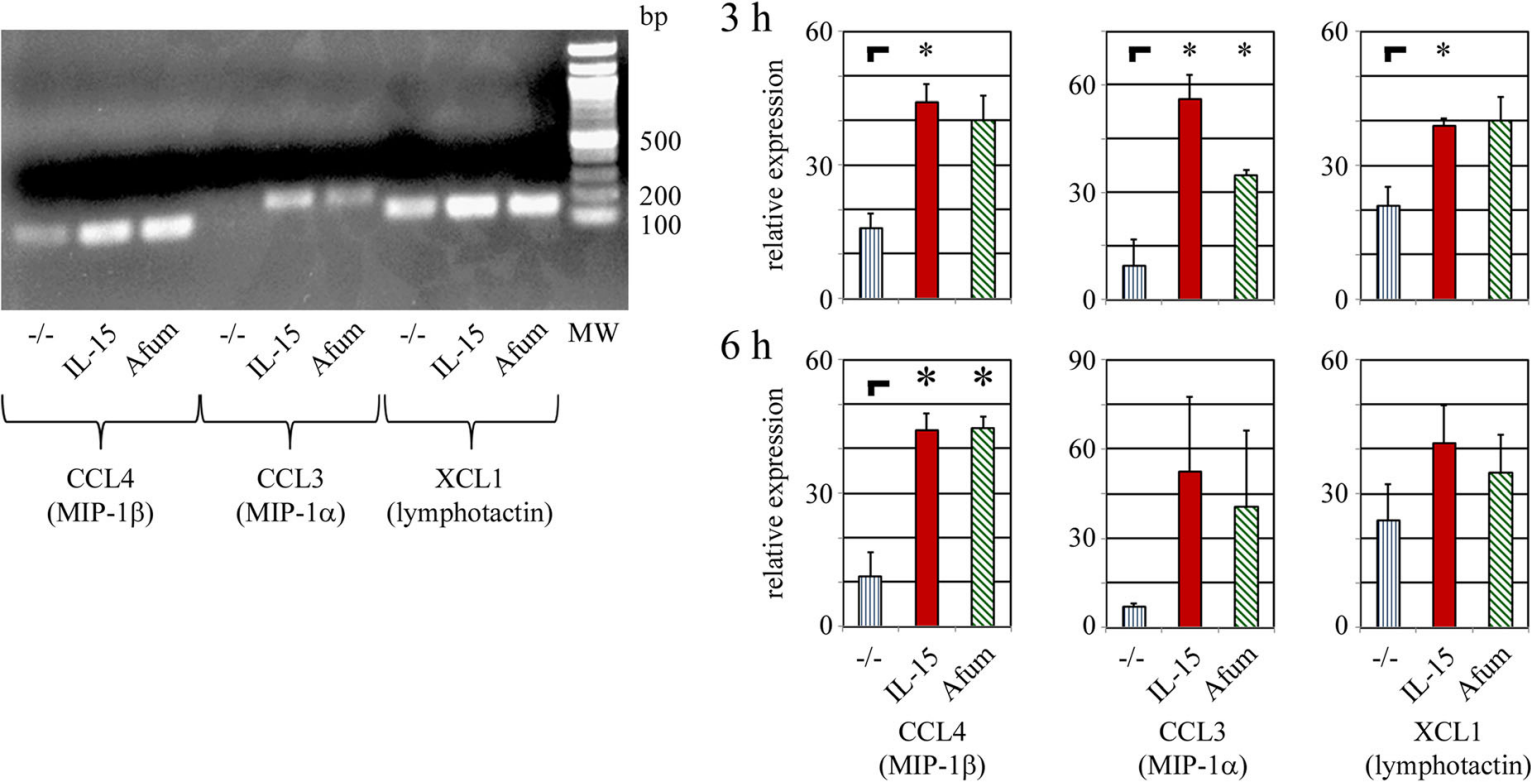
Expression of cytokine mRNA after 3 and 6 h of stimulation analyzed by conventional PCR. Left side: Freshly isolated NK cells were pre-incubated overnight with pro-leukin and stimulated with IL-2 / Il-15 (IL-15) or *A. fumigatus* (Afum) germ tubes for 3 h. Unstimulated controls were included (−/−). RNA was isolated and used for RT-PCR detection of CCL4/MIP-1β, CCL3/MIP-1α, and XCL1/lymphotactin expression. PCR products were analyzed by gel electrophoresis. Right side: The experiment was performed three times in total for 3 (upper row) and 6 h (lower row) of stimulation, as indicated. Using ImageJ, the relative color intensity of each DNA band was quantified as a measure for the initial expression rate of the appropriate mRNA. Means and standard deviations were summarized in diagrams, *p < 0.05

In the qPCR analysis, relative cytokine messenger RNA (mRNA) expression was calculated by normalization to 5′-aminolevulinate synthase 1 (ALAS1) expression levels. Thereby, factors as (due to stimulation) additionally produced mRNAs or the real number of NK cells before/during/after stimulation were intentionally taken out of calculation. Now, in order to visualize the situation considering these factors, corresponding PCR fragments for CCL3/MIP-1α, CCL4/MIP-1β and XCL1/lymphotactin were analyzed by gel electrophoresis. Consistently, subsequent densitometric evaluations were performed without including ALAS1 for normalization (Fig. 2). The expression levels of all three tested chemokines were upregulated in NK cells already after 3 h stimulation, with just a marginal difference to the expression levels after 6 h. Due to the normalization mentioned above, qPCR analysis revealed a higher expression level for CCL3/MIP-1α and CCL4/MIP-1β after *A. fumigatus* versus (vs.) IL-2/IL-15 stimulation. Here, a densitometric evaluation detects the same expression level for both stimulations, or even a higher expression level for IL-2/IL-15 stimulation.

### Release of CCL4/MIP-1β

**Fig. 3:**
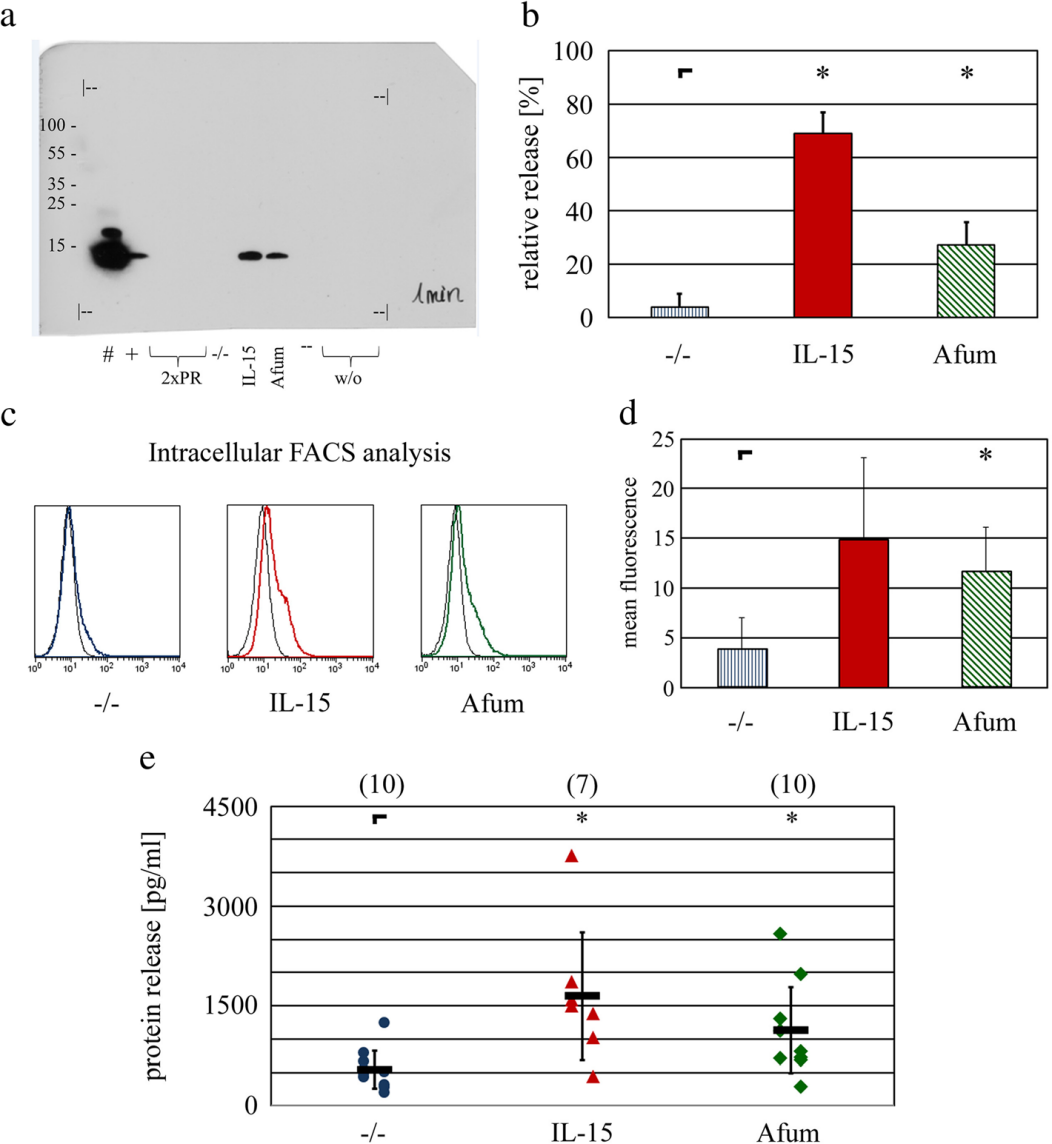
Release of CCL4/MIP-1β by stimulated NK cells. NK cells were prepared and stimulated as described in methods. **a** Supernatants were incubated with αCCL4/MIP-1β and A/G sepharose beads, culture medium was included as negative control (−). Immunoprecipitated proteins were analyzed by SDS page and subsequent Western Blot for CCL4/MIP-1β protein. 100 ng (#) and 100 pg (+) rhCCL4/MIP-1β were provided as positive controls. Two lanes were used for molecular weight standards (PR), results are indicated in kDa on the very left. Another two lanes were not used (w/o). For your convenience, the whole X-ray film is shown, the size of the membrane is marked with “|--”. Please note the exposition time on the right corner (1 min) and the appearance of high molecular weight aggregates within the overloaded positive control (#). **b** The experiment described in (**a**) was performed four times in total and analyzed by ImageJ. Means and standard deviations were summarized into one diagram. **c** Cytokine release was blocked by Brefeldin A. CCL4/MIP-1β expression was analyzed by intracellular FACS analysis (colored, thick lines) including isotype controls (thin, black lines) as shown here in a representative result. **d** The experiment described in (**c**) was performed three times in total. MFI values were corrected as described in the method section, means and standard deviations were summarized into one diagram. **e** Supernatants of stimulated NK cells were analyzed by ELISA for CCL4/MIP-1β. The number of samples is indicated for each stimulation condition. Individual results as well as means and standard deviations are depicted. *p < 0.05

In Fig. 2, expression increase of CCL4 – but not of CCL5 or XCL1 – after 6 h stimulation with *A. fumigatus* is not only significant, but also on a similar level as the expression increase after IL-15 stimulation. Additionally, CCL4/MIP-1β has already been shown to reveal higher concentrations in the supernatant of stimulated NK cells than 24 other tested cytokines – including IFN-γ, TNF-α, CCL3/MIP-1α and CCL5/RANTES [20]. Therefore, CCL4/MIP-1β was expected to be detected easier than other cytokines and was exemplarily selected for further analysis of protein release by different methods (Fig. 3). Freshly isolated NK cells were stimulated as mentioned above, the supernatant was analyzed by immunoprecipitation and subsequent Western Blot analysis for the presence of CCL4/MIP-1β. A protein band of similar size as the positive control (approximately 7.8 kDa) was detected in the immunoprecipitate of the supernatants from stimulated, but not from unstimulated, NK cells. Additionally, the Western Blots from four different experiments were densitometrically evaluated. In line with the results from Figs. 1 and 2, NK cells showed a significant higher release of CCL4/MIP-1β after stimulation with IL-2/IL-15 or *A. fumigatus*, while the CCL4/MIP-1β concentration was highest after stimulation with IL-2/IL-15 (Fig. 3a and b).

In order to further corroborate the release of CCL4/MIP-1β, intracellular fluorescence-activated cell scanning (FACS) analysis was performed. Freshly isolated NK cells were stimulated as described above, but additionally had Brefeldin A added after 1 h of stimulation. After 6 h of stimulation in total, cells were collected, fixed, permeabilized and finally stained with α-CCL4/MIP-1β and a fluorochrome-conjugated second step antibody (Fig. 3c). CCL4/MIP-1β was detected to a minor extent in unstimulated, on a higher level with *A. fumigatus* stimulated, and clearly visible with IL-2/IL-15 stimulated NK cells. Three different experiments were taken into account for a statistical approach (Fig. 3d). In line with former results, expression of CCL4/MIP-1β was increased due to stimulation to a higher extent by IL-2/IL-15 vs. *A. fumigatus*.

Finally, supernatants of stimulated and unstimulated NK cells were analyzed for CCL4/MIP-1β by enzyme-linked immunosorbent assays ELISA (Fig. 3e). The release of CCL4/MIP-1β was significantly increased by stimulation with IL-2/IL-15 or *A. fumigatus*.

### Cytokine production and release are adjusted to the MOI of *A. fumigatus*

**Fig. 4:**
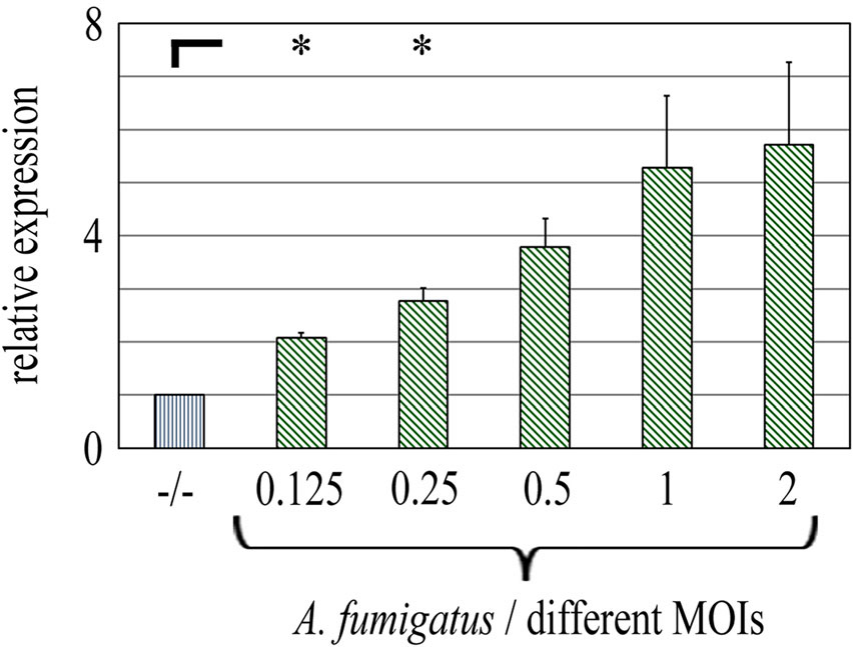
Expression of CCL4 (MIP-1β) mRNA by NK cells after stimulation with different MOIs of *A. fumigatus*. NK cells were stimulated with increasing MOIs of *A. fumigatus* as indicated or left unstimulated (−/−) for 6 h. Expression of CCL4 mRNA was examined by qPCR. *n* = 3. *p < 0.05

In order to elucidate a potential dose dependent effect on the cytokine release, NK cells were stimulated as described above but with increasing MOIs of *A. fumigatus*. In order to get a first impression, expression of CCL4 mRNA was found to increase dose dependently according to the MOI (Fig. 4).

**Fig. 5:**
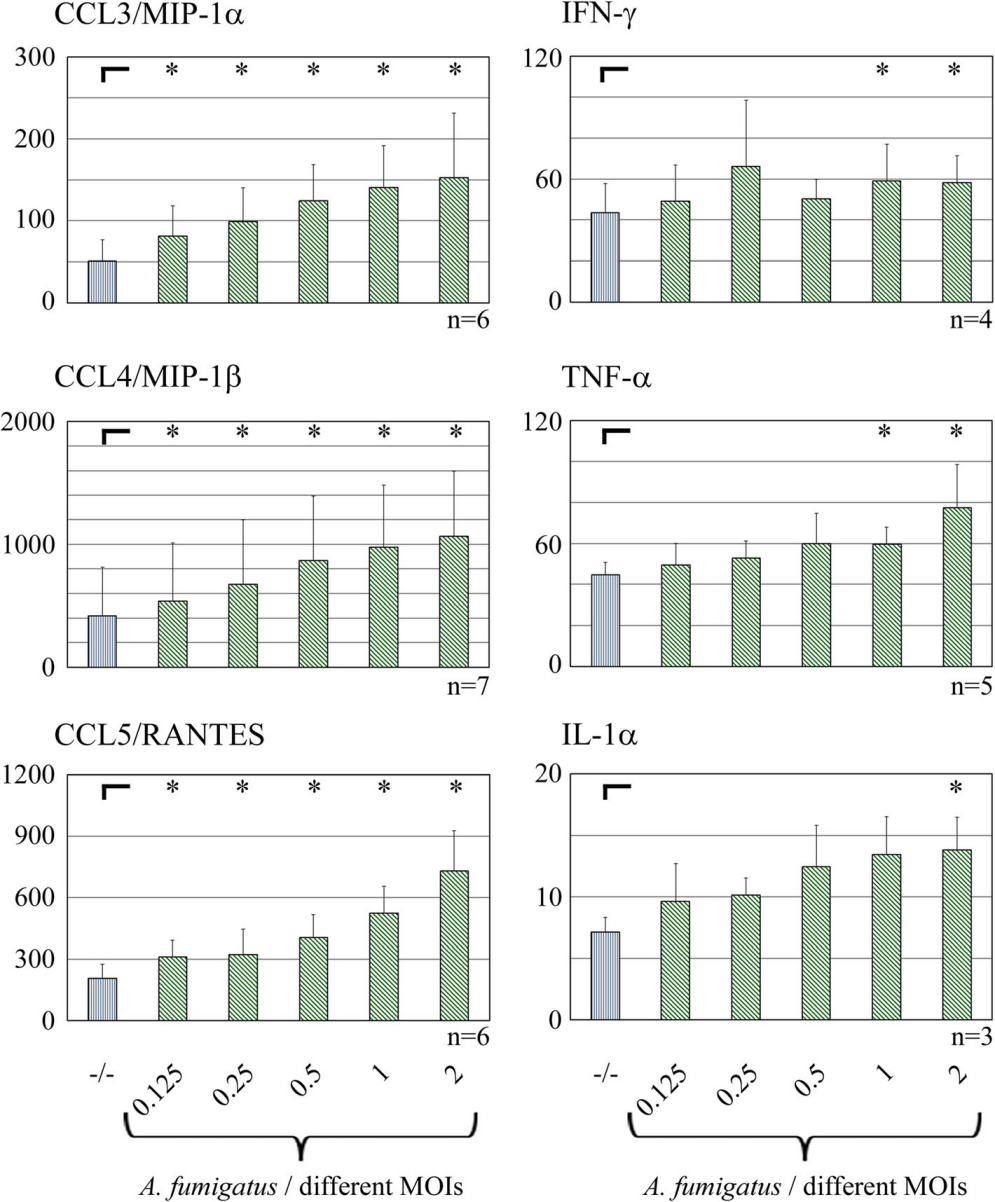
Release of cytokines by NK cells after stimulation with different MOIs of *A. fumigatus.* NK cells were stimulated with increasing MOIs of *A. fumigatus* as indicated or left unstimulated (−/−) for 6 h. The release of cytokines was analyzed by ELISA. All measurements are noted in pg/ml, the number of independent experiments for every cytokine is indicated. *p < 0.05

Subsequently, cytokine release was analyzed by ELISA (Fig. 5). Chemokines such as CCL3/MIP-1α, CCL4/MIP-1β and CCL5/RANTES showed a significant stepwise increase up to 1000 (CCL4/MIP-1β) pg/ml referring to an increasing MOI. The cytokines TNF-α and IL-1α revealed a gradually increasing release, as well, but – presumably due to low absolute concentrations – showed significant difference to the release of unstimulated NK cells only with the highest MOIs. IFN-γ release was increased in general, but did not show a continuous rise according to the MOIs. Taken together, protein releases of all cytokines but IFN-γ were gradually amplifying due to increasing MOIs.

### CD56 availability and CD69 expression on NK cells reflect the MOI of *A. fumigatus*

**Fig. 6:**
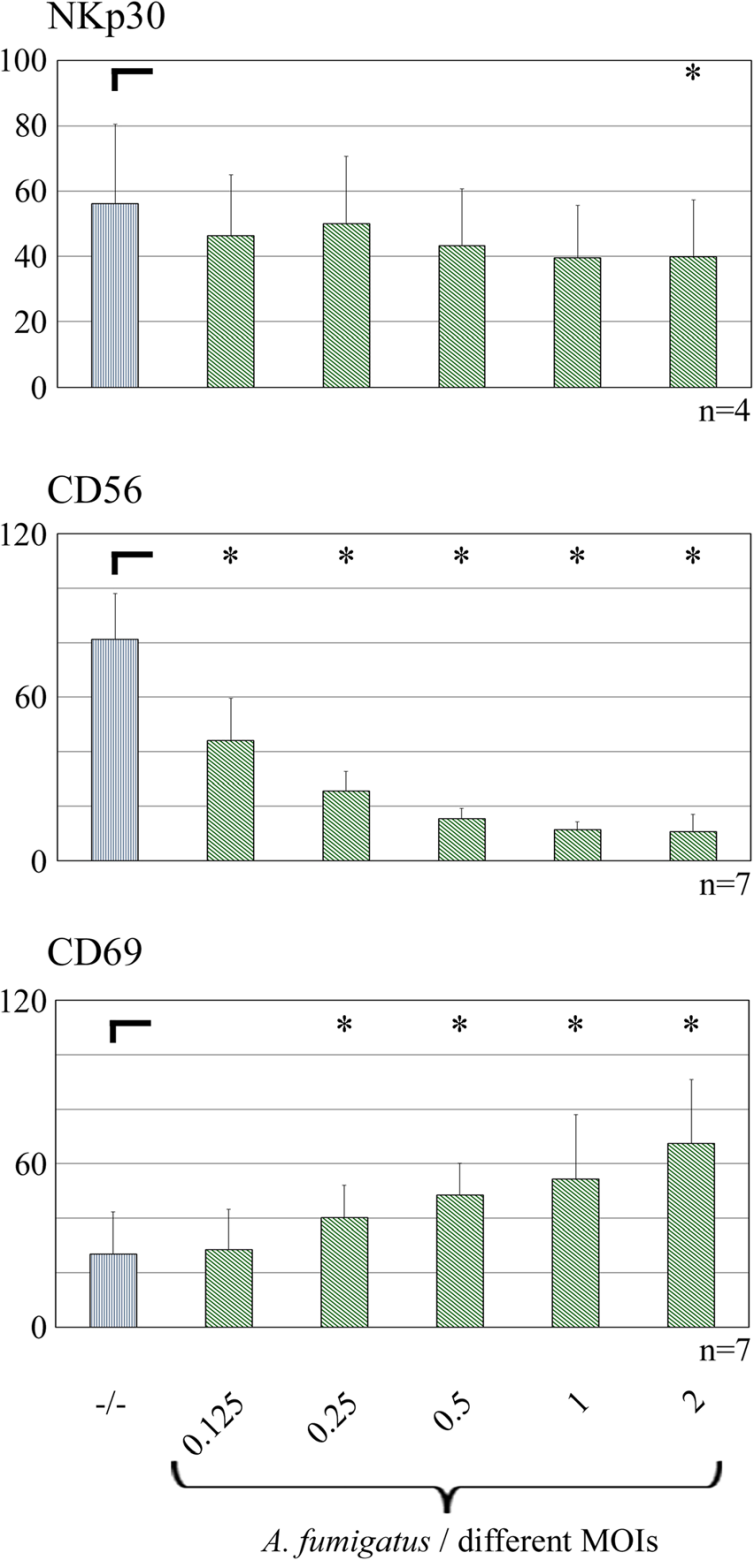
Expression / availability of receptors on NK cells after stimulation with different MOIs of *A. fumigatus.* NK cells were stimulated with increasing MOIs of *A. fumigatus* as indicated or left unstimulated (−/−) for 6 h. NKp30, CD56 and CD69 were examined by FACS analysis. Mean fluorescence intensity values are shown. The number of independent experiments for every cytokine is indicated. *p < 0.05

While this study was in preparation, our group published data revealing CD56 as a pathogen recognition receptor (not only) for *A. fumigatus*. In line with these results, we verified the gradually decrease of CD56 availability on the surface of NK cells after stimulation with the MOIs used in the present study. Additionally, CD69 and NKp30 expressions were examined (Fig. 6). As expected, CD69 expression increased significantly due to higher MOIs. NKp30 expression, on the other hand, was barely influenced by the presence of *A. fumigatus*.

## Discussion

In clinical practice, *A. fumigatus* infections in immunocompromised patients are an increasing problem. NK cells are more and more considered as a mandatory factor for the outcome, since they are known for the release of a wide spectrum of cytokines and thereby intended to play a major role in the early phases of an immune response. In this study, the expression and release of cytokines by freshly isolated human NK cells after *A. fumigatus* stimulation were analyzed. Cytokine expression by (q)PCR and analysis of protein release by ELISA and – for CCL4/MIP-1β – by complimentary experiments including Western Blot and FACS analysis were evaluated. Additionally, the release of chemokines, cytokines, and the expression of surface markers by NK cells after stimulation with different MOIs of *A. fumigatus* were examined.

**Chemokines** such as CCL3/MIP-1α and CCL4/MIP-1β were most prominently upregulated in stimulated NK cells, while CCL5/RANTES showed a slight downregulation on mRNA level, but a marked increase on protein release. Furthermore, the chemokine concentration was correlated with the MOI of *A. fumigatus*.

The expression and – in part – the release of cytokines by other immune cells after confrontation with *A. fumigatus* is already known. Since CCL3/MIP-1α, CCL4/MIP-1β and TNF-α mRNA expression in dendritic cells (DCs) or monocytes is upregulated after co-incubation with *A. fumigatus*, these results are in line with the present study. On the contrary, the analysis of CCL5/RANTES expression and release by further immune cells shows inconsistent results. While the mRNA is upregulated in DCs, the corresponding protein is hardly detectable after 20 h of co-cultivation with *A. fumigatus*. In monocytes, mRNA expression and the release of CCL5/RANTES is even downregulated [35–39]. Platelets, on the other hand, show an increase of CCL5/RANTES protein after *A. fumigatus* stimulation [40]. It should be noted, that CCL5/RANTES expression in T cells was intensively analyzed around 20 years ago, mainly due to its anti-HIV effect, but also due to the characteristically late expression of the CCL5 gene [41].

Beside that particular CCL5 mRNA expression characteristic, there are other possible mechanisms to explain the results of the present study. For example, resting murine NK cells contain low amounts of perforin and granzyme B proteins, while the corresponding mRNAs are abundant. Upon activation, the available mRNAs are translated, while there are just slight changes to the amount of mRNAs available [42]. It is tempting to speculate, that there may be a similar mechanism for CCL5/RANTES. Alternatively, NK cells may simply empty their granules upon *A. fumigatus* recognition, but further focus on the expression of cytokines other than CCL5/RANTES for acute treatment of the fungal infection. It may be of interest for further studies, whether, and if so when, the expression level of CCL5/RANTES is increasing again after the stimulation has taken place.

In addition, genetic studies revealed single nucleotide polymorphisms (SNPs) in CCL3 and CCL4, which are significantly correlated to fungal infections [43]. In particular, CCL3/MIP-1α is regarded as a critical mediator of host defense against *A. fumigatus* infection due to several studies of animal infection models [44–47]. Together with CCL4/MIP-1β and CCL5/RANTES, CCL3/MIP-1α is responsible for the recruitment of various immune cells such as monocytes, T cells, all kinds of granulocytes, DCs and NK cells [48–51]. Therefore, these chemokines might play a substantial role in orchestrating an early antifungal response against *A. fumigatus*.

**Cytokines** such as IFN-γ, TNF-α and IL-1α revealed a vast upregulation on mRNA level and – except for IFN-γ – a slight but stepwise increase on the protein release when stimulated with different MOIs of *A. fumigatus*. Even though IFN-γ release showed a significant upregulation after stimulation with an MOI of 1 or 2, the respective gain of IFN-γ did not fully correspond to the stepwise increase of the MOI. That may be due to the combination of different characteristics of IFN-γ release by NK cells. First, cytokine vs. chemokine release by stimulated NK cells generally reveals a greater requirement for receptor cooperation and, probably therefore, occurs at later time points after stimulation [20]. Second, the release of IFN-γ by NK cells after 6 h of *A. fumigatus* stimulation is very low in general as seen in the present and in a preceding study by Bouzani et al. In the latter publication, IFN-γ release was shown to reach a peak not until 12 h of incubation [27]. Third, according to Schneider et al., *A. fumigatus* actively impairs the release of IFN-γ (and GM-CSF) by NK cells [34]. Taken together, these effects could be responsible for the inconsistent results of the MOI-dependent analysis of IFN-γ release by stimulated NK cells.

IFN-γ is a main activator of macrophages and cytotoxic T cells, and favors T helper cell type 1 immune responses. Former studies have already demonstrated beneficial effects of IFN-γ against *A. fumigatus* infection [26, 52–54]. Moreover, Bouzani et al. showed that NK cell-derived IFN-γ has a direct antifungal activity. In line with the results of the present study, IFN-γ mRNA expression was already detectable after 6 h, while IFN-γ protein was just slightly released after 6 h, but substantially present in the supernatant of NK cells after 12 h of stimulation. In contrast, TNF-α mRNA expression and protein release could be detected already after 6 h [27] – again in line with results of this study. Furthermore, genetic studies show a correlation between SNPs in the genes for IFN-γ (IFNG) and tumor necrosis factor receptor 2 (TNFR2) and the risk of suffering from a disease caused by *A. fumigatus* [55].

IL-1α is a major cytokine to induce and regulate inflammation in general. With regard to antifungal effects, IL-1α was described as crucial for optimal leukocyte recruitment during a pulmonary *A. fumigatus* infection [56], identified as an inductor for antimicrobial peptide expression in epithelial cells [57] and shown to be a main coordinator in the neutrophil response to *Candida albicans* [58]. IL-1α was found to be effective already at concentrations of about 10 pg/ml [59], which is lower than the IL-1α concentration found in this study. Taken together, the upregulation of IFN-γ mRNA expression, which – according to Bouzani et al. [27] – will likely be followed by an upregulated protein release later on, and the increase of TNF-α and IL-1α release demonstrated in this study can be regarded as substantial contribution to an immune response against *A. fumigatus*.

With regard to the comparison of chemokine (CCL3/MIP-1α, CCL4/MIP-1β) vs. cytokine (IFN-γ, TNF-α) release by stimulated NK cells, Fauriat et al. have already characterized the cytokine secretion profile of NK cells after contact with K562. While chemokines such as CCL3/MIP-1α, CCL4/MIP-1β and CCL5/RANTES were detected right after stimulation, cytokines such as TNF-α and IFN-γ were found only later on [20]. These results are in line with data published by Bouzani et al., showing a vast increase of IFNG and TNF expression and – only for TNF-α – a relatively moderate increase of protein release after 6 h. After 12 h, though, IFN-γ and TNF-α release showed a strong and significant increase after stimulation with *A. fumigatus*. In line with these publications, IFN-γ and TNF-α secretions after just 6 h stimulation in the present study were very low. Thereby, a fine-tuning of the release by stepwise increasing MOI was just recognizable in an initial stage. At this early time point after stimulation, relatively high MOIs of 1 or 2 are needed for the initiation of a significantly increased release of the cytokines. NK cells seem to stepwise counteract a fungal infection with recruitment of macrophages and neutrophils in the early phases, while adaptive immunity will be alarmed by cytokines in case the infection persists.

Very recently, Schneider et al. analyzed cytokine expression by NK cells after stimulation with *A. fumigatus*, as well [34]. In accordance with the present study, that publication reported a downregulation of GZMB (granzyme B) and an upregulation of IFNG (IFN-γ), CSF2 (GM-CSF), CCL3 (MIP-1α) and CCL4 (MIP-1β) mRNA expression, and an increase of the intracellular protein level of IFN-γ after *A. fumigatus* stimulation. In contrast to the results obtained here, subsequent analysis of the supernatant revealed a non-significant downregulation of IFN-γ, CCL3/MIP-1α or CCL4/MIP-1β release. That may be explained by the different pre-treatment of the NK cells used in both studies. Schneider et al. focused their research on a clinical background and therefore pre-stimulated NK cells over 10 days with the addition of 1000 U/ml IL-2 every three days, before the cells were co-incubated with *A. fumigatus*. The authors discuss that procedure with regard to perforin release, revealing that IL-2 pre-stimulation of NK cells alone already results in relatively high extracellular perforin levels, and that – presumably therefore – *A. fumigatus* had only a marginal additional effect on the extracellular perforin concentration. It is tempting to speculate, that this is valid not only for perforin, but also for cytokine production in general, and that IL-2 pretreatment only overnight – as in our study – just ignite NK cell activity and thereby leaves room for an increase of IFN-γ, CCL3/MIP-1α and CCL4/MIP-1β release after *A. fumigatus* stimulation.

The **surface markers** NKp30 and CD56 were reported as binding receptors for fungal ligands in former publications. Li et al. pretreated NK cells with increasing amounts of a preparation of the cryptococcal cell wall/membrane and subsequently showed a decreasing accessibility of NKp30 by an adequate antibody [30]. Here, the surface availability of NKp30 decreased just roughly according to increasing MOIs of *A. fumigatus*, but showed a significant difference in comparison of unstimulated vs. NK cells after stimulation with the highest MOI. As expected due to results by Ziegler et al. [29], the accessibility of CD56 on the cell surface decreased significantly and stepwise according to increasing MOIs. In turn, that indicated a gradually increased amount of CD56 molecules engaged in the binding process. Moreover, a correlation between CD56 binding activity and the activation status reflected by CD69 expression depending on different MOIs of *A. fumigatus* was shown.

The **correlation of the** ***A. fumigatus*** **MOI** with the cytokine expression and release was described here – at least to our knowledge – for the first time. NK cells seem to fine-tune their immunological reactions according to the threat level, thereby not only providing information about the mere presence, but also about the relative amount of *A. fumigatus*. Future studies will analyze whether this feature of NK cells can be shown as well for the expression and release of other immunomodulatory molecules and/or toward further pathogens. Additionally, the relatively simple comparative measurement of chemokine expression by qPCR, chemokine release by ELISA or CD69 expression by FACS analysis may become an instrument to quantify the involvement of NK cells in an ongoing immune reaction.

## Conclusions

In summary, we corroborate the results from Li et al. [30] and Ziegler et al. [29] with regard to the surface markers, but further provide evidence for an MOI-dependent activation status of and a cytokine release by NK cells after stimulation with *A. fumigatus*. The contribution of NK cells to the immune response against *A. fumigatus* infection may therefore be based upon their early intervention in the cytokine network. Since the dose-dependent release of CCL4/MIP-1β provides relatively high concentrations in a short period after stimulation, it may be regarded as a marker protein for NK cell activation in further studies. These results will contribute to our understanding of the interactions between NK cells and *A. fumigatus*, which may be critical in patients suffering from IA while recovering from HSCT.

## Methods

### NK cell purification and stimulation

Peripheral blood mononuclear cells (PBMCs) were isolated by Ficoll (Biochrom AG) density gradient centrifugation using leukoreduction system chambers obtained from healthy adult blood donors. Usage of the human blood specimens was approved by the Ethical Committee of the University Hospital Wuerzburg, and written consent was provided by all blood donors. NK cells were isolated by magnetic-activated cell separation (MACS) NK negative selection kit (Miltenyi Biotec). The purity was checked by FACS analysis using CD14-Fitc, NKp46-PE, CD56-APC (all from BD Pharmingen) and CD3-PerCP (Miltenyi Biotec) antibodies, and was always > 95% (please see Additional file 1). NK cells were cultured overnight in RPMI 1640 (Invitrogen) with 10% heat-inactivated fetal bovine serum, 120 μg/ml gentamicin (Refobacin; Merck) and supplemented with 1000 U/ml recombinant human IL-2 (Novartis) at 37 °C and 5% CO_2_. *A. fumigatus* (ATCC 46645) germ tubes were prepared as described previously [37] and added to NK cells at an MOI of 0.5 unless indicated otherwise. For comparison, NK cells were left unstimulated or – for usage as positive control – added IL-2 (500 U/ml) and IL-15 (500 U/ml = 62.5 ng/ml), which stimulate for NK cell activation [60, 61]. While preceding studies using *A. fumigatus* conidia for stimulation on macrophages or epithelial cells revealed a minimum incubation time of about 8 h to take effect [39, 62], studies using already prepared germlings provide an incubation time of around 6 h as adequate for a subsequent simultaneous analysis of mRNA and protein expression for early expressed genes [27]. Therefore, the latter method was chosen, while partially additional samples were collected after 3 h. Cells were used for RNA isolation or FACS analysis, while the supernatants were submitted to ELISA or immunoprecipitation of CCL4/MIP-1β with subsequent Western Blot analysis.

### Gene expression analysis

RNA isolation was performed using RNeasy Mini Kits (Qiagen), while subsequent cDNA synthesis was done using First Strand cDNA Synthesis Kit (Thermo Fisher), each according to the manufacturer protocol. Nucleic acid concentration was determined by nanodrop quantification (Thermo Scientific). qPCR analysis was processed using primers (Table 1) from Sigma-Aldrich and SYBRGreen Master Mix from Biorad in a Step One Plus (Applied Biosystems).

**Table 1:** Primers for qPCR analysis

Gene	Forward	Reverse	Product size (bp)
ALAS1^a^	GGCAGCACAGATGAATCAGA	CCTCCATCGGTTTTCACACT	150
CCL3 (MIP-1α)	TGCAACCAGTTCTCTGCATC	TTTCTGGACCCACTCCTCAC	198
CCL4 (MIP-1β)	GCTTCCTCGCAACTTTGTGG	TCACTGGGATCAGCACAGAC	111
CCL5 (RANTES)	TCATTGCTACTGCCCTCTGC	TACTCCTTGATGTGGGCACG	115
CXCL8 (IL-8)	GGTGCAGTTTTGCCAAGGAG	TTCCTTGGGGTCCAGACAGA	183
XCL1 (lymphotactin)^b^	CTCCTTGGCATCTGCTCTCT	CCTTCCGTGATGGTGTAGGTC	137
IFNG (IFN-γ)^c^	GCATCCAAAAGAGTGTGGAG	GCAGGCAGGACAACCATTAC	255
TNF (TNF-α)	TGCTTGTTCCTCAGCCTCTT	TGGGCTACAGGCTTGTCACT	185
CSF2 (GM-CSF)	GCCCTGGGAGCATGTGAATG	CTTGTAGTGGCTGGCCATCAT	223
IL1A (IL-1α)	TGATCAGTACCTCACGGCTG	TGGTCTTCATCTTGGGCAGT	156
IL16 (IL-16)	CGAAGACTCAGCTGCAAATGG	GCAGGGAGATAACGGACTGAC	167
GZMB (granzyme B)	TGCGAATCTGACTTACGCCA	GCATGCCATTGTTTCGTCCA	160

^a^ALAS1 is well established as a housekeeping gene for the normalization of gene expression by cells confronted with different stimuli [63–67]. In general, primers were designed to include as much transcript variants of the mentioned gene as possible. Within this context, ^b^primers for XCL1/lymphotactin qualify also for the amplification of XCL2, which differs just slightly with regard to the amino acids, but show the same functionality in vitro [68]. ^c^Primers for IFNG were already published by Bouzani et al. [27]

Expression of every gene was normalized to the expression of the housekeeping gene ALAS1. Relative expression of the respective gene in unstimulated NK cells was converted to “1”, its expression level in stimulated NK cells was calculated in correlation to that.

PCR amplicons of CCL3 (MIP-1α), CCL4 (MIP-1β) and XCL1 (lymphotactin) were submitted to agarose (Roth) gel electrophoresis, using ethidium bromide from Thermo Fisher and an electrophoresis system from Serva. The results were documented by a Multi-Image Light Cabinet (Alpha Innotech) and densitometrically analyzed by ImageJ. The intensities of bands generated by stimulated and non-stimulated NK cells of one experiment were added up und accounted for 100%. For every stimulation condition, the respective percentage was calculated.

### Quantification of released CCL4/MIP-1β by immunoprecipitation and Western blot

0.5 μg monoclonal mouse anti human CCL4/MIP-1β antibody (R&D Systems) was pre-incubated with 15 μl Protein A/G Plus Agarose Beads (Santa Cruz Biotechnology) for 3 h at 4 °C, then added for overnight incubation to the NK cell supernatants generated above. After that, the beads were submitted for running on a 15% sodium dodecyl sulfate (SDS) gel, transferred to a nitrocellulose membrane and blotted with 0.1 μg/ml polyclonal biotinylated anti-CCL4/MIP-1β (R&D Systems). After 2 h at 4 °C, the membrane was supplemented with Streptavidin-HRP (Biolegend) and finally developed using ECL chemiluminescence reagent. Recombinant human CCL4/MIP-1β (R&D Systems) was included as positive control. The X-ray film was scanned and densitometrically analyzed by ImageJ as described above.

### Intracellular detection of CCL4/MIP-1β by FACS analysis

NK cells were isolated and initially stimulated as mentioned above, but supplemented with 10 μg/ml Brefeldin A after 1 h of incubation with *A. fumigatus*, IL-2/IL-15 or nothing. After 6 h in total, cells were permeabilized (Cytofix/Cytoperm Kit / BD Biosciences) and intracellularly stained with monoclonal mouse anti human CCL4/MIP-1β or IgG Isotype control (both R&D Systems), and subsequently treated with PE-conjugated anti mouse antibody (Jackson Immunotech). All samples were measured on a FACS Calibur flow cytometer (BD Biosciences) using the Cell Quest Pro software. The mean fluorescence intensity (MFI) obtained by the isotype control was subtracted from the respective MFI obtained by the CCL4/MIP-1β analysis in order to receive a corrected MFI for further evaluation.

### Quantification of released molecules by ELISA

The concentration of immunomodulatory molecules were quantified using ELISA Kits from R&D Systems (CCL3/MIP-1α, CCL4/MIP-1β, IFN-γ) and Biolegend (CCL5/RANTES, IFN-γ, TNF-α and IL-1α) according to the manufacturer’s protocol.

### Expression of surface molecules by FACS analysis

αNKP30-PE (Biolegend), αCD56-Fitc (Becton Dickinson), αCD69-APC (Miltenyi) and respective isotype controls from the same companies were used for the analysis of the surface expression.

### Statistical analysis

In figures, n refers to the number of independent experiments. Statistical significance was tested for results obtained from unstimulated vs. IL-2/IL-15 stimulated (“IL-15”) or vs. *A. fumigatus* stimulated (“Afum”) NK cells, respectively, using student’s t-test. Therefore, in the presentation of the figure, results from unstimulated NK cells were marked with ⌐ for being the standard, while results from stimulated NK cells were marked with * (*p* < 0.05) or nothing (*p* ≥ 0.05).

## Additional file

Additional file 1: **Figure S1.** Dot Plot visualizing the purity of NK cells after negative isolation by MACS. PBMCs before (PBMC) and after (PBMC w/o NK cells) NK cell isolation were analyzed for NKp46 (NK cells), CD14 (MØ), CD3 (T cells) and CD19 (B cells) expression. Isolated NK cells were also included in the analysis before subsequent experiments. One representative result is shown, results were rounded to the nearest whole number. Purity of isolated NK cells was always above 95%. (PDF 1454 kb)

## Abbreviations

(m)RNA: (messenger) ribonucleic acid
(q)PCR: (quantitative) polymerase chain reaction
*A*.: *Aspergillus*
ALAS1: 5′-aminolevulinate synthase 1
CCL: CC chemokine ligand
CD: Cluster of differentiation
CXC3L1: Chemokine (C–X3–C motif) ligand 1
DC: Dendritic cell
ELISA: Enzyme-linked immunosorbent assay
FACS: Fluorescence-activated cell scanning
GM-CSF: Granulocyte-macrophage colony-stimulating factor
h: Hours
HSCT: Hematopoietic stem cell transplantation
IA: Invasive aspergillosis
IFN: Interferon
IL: Interleukin
IP-10: N-gamma-inducible protein-10
MACS: Magnetic-activated cell separation
MCP-1: Monocyte chemoattractant protein 1
MFI: Mean fluorescence intensity
MIP: Macrophage inflammatory protein
MOI: Multiplicity of infection
PBMC: Peripheral blood mononuclear cells
RANTES: Regulated and normal T cell expressed and secreted
SDS: Sodium dodecyl sulfate
SLC: Secondary lymphoid tissue chemokine
SNP: Single nucleotide polymorphism
TLR: Toll-like receptor
TNF: Tumor necrosis factor
vs.: Versus
XCL1: X-C motif chemokine ligand 1
