# Supplementary figures and images for: Human NK cells adapt their immune response towards increasing multiplicities of infection of Aspergillus fumigatus
Source: BMC Immunol. 2018 Dec 18;19:39. doi: 10.1186/s12865-018-0276-6 (PMC6299526; doi:10.1186/s12865-018-0276-6)

PBMC

CD14

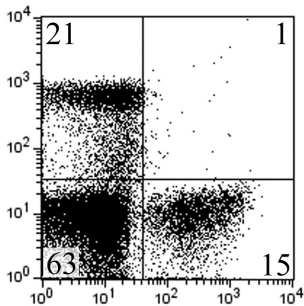

CD3

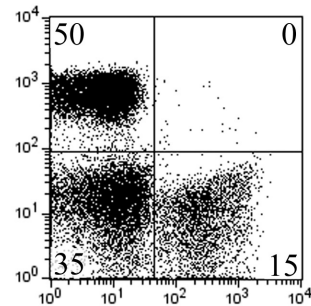

CD19

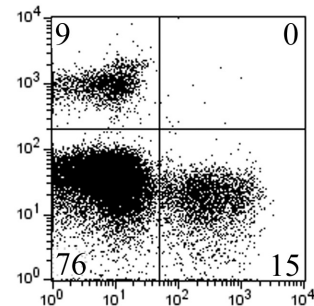

PBMC  
w/o  
NK cells

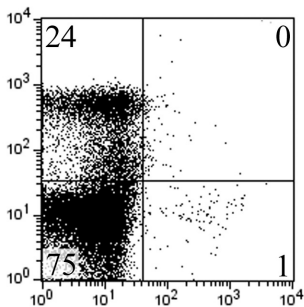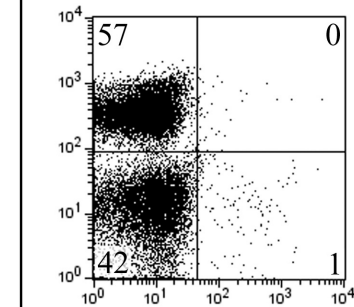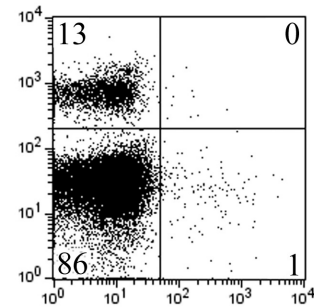

NK cells

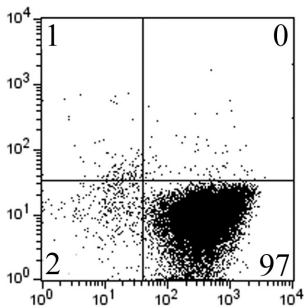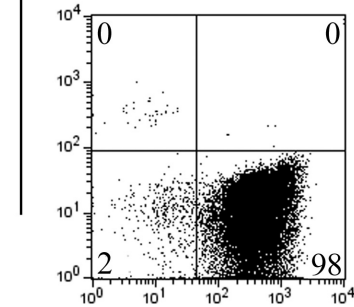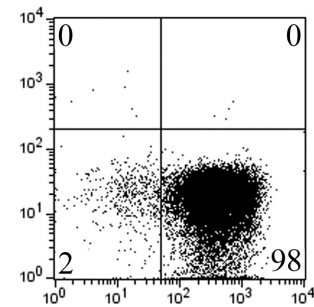

NKp46

Supplement: Supplementary file 1 — Figure S1. Dot Plot visualizing the purity of NK cells after negative isolation by MACS. PBMCs before (PBMC) and after (PBMC w/o NK cells) NK cell isolation were analyzed for NKp46 (NK cells), CD14 (MØ), CD3 (T cells) and CD19 (B cells) expression. Isolated NK cells were also included in the analysis before subsequent experiments. One representative result is shown, results were rounded to the nearest whole number. Purity of isolated NK cells was always above 95%. (PDF 1454 kb) [file 12865_2018_276_MOESM1_ESM.pdf]
